# Insanity in India
*Practical Remarks on Insanity in Bengal. By Thomas N. Wise, M.D., late Surgeon H. E. I. C. Service.


**Published:** 1853-07-01

**Authors:** 


### Art. III.—INSANITY IN INDIA.*

There is, perhaps, no country which presents a more extensive field for psychological observation, nor which offers to the mental pathologist more abundant materials for induction, than that of India. It is, however, deeply to be regretted that the labourers in this field hitherto have been few, and that as yet no work on the subject, alike comprehensive and systematic, has issued from the press. The author of the brochure before us must have had, as medical superintendent of the Lunatic Asylum at Dacca, very favourable opportunities for studying insanity in a section of our Indian colony. Many of the facts which he has published, are full of interest; he has, however, been led into error, by making hasty deductions from necessarily imperfect statistical data, and has also advanced some views which are not only unsubstantiated by facts, but even contradictory. We require much more research, ere a correct knowledge of the various phases of insanity, as they are developed in India, can be arrived at.

It would take more space than this journal could afford, at the present time, to give other than a hasty allusion to the various momentous topics which are suggested at the mere mention of the name of India. We need only refer to her remains of ancient grandeur, her high state of civilization before the conquest of Alexander, her peculiar religion and unequalled fanaticism, the singular division of her people into castes and their highly intellectual capacity, the fearful ravages to which she has from time to time been subjected by the sword, pestilence, and famine, to show the importance of the subject, as well as to establish the fact, that all the known predisposing causes of insanity have been in operation for centuries, and that Dr. Wise, consequently, takes a very partial view of the subject, when he infers (though, by the way, he subsequently contradicts himself) that the prevalence of insanity in India, is owing to the illiterate condition of the people. But did we admit the fact, that ignorance is to be ranked among the causes of insanity, which we do not, no one acquainted with the history of India can agree with him that the Hindoos are "perhaps in a lower state of mental development than even the rudest savage."

He affirms that, "although intimately connected with the educational department of the Bengal government, during a long series of years, I never knew of a well-educated native becoming insane." This experience is not borne out by the observations of our best authorities, who, on the contrary, find that insanity frequently, perhaps *most* frequently,

* Practical Remarks on Insanity in Bengal. By Thomas N. Wise, M.D., late Surgeon H. E. I. C. Service.

attacks the refined and mentally cultivated. In our consideration of the causes which are likely to engender that peculiar condition of the brain, whatever it may be, on which the predisposition to insanity depends, and which are in operation in our Indian empire, we must not forget the excitement of their superstitious idolatry, and of that unnatural fanaticism so closely allied to insanity ; a fanaticism so intense that it leads the miserable devotees to practise the most cruel tortures upon their own bodies. Nor must we lose sight of the high development of the imaginative faculty, as evinced in the ancient poetry of India, and in her gigantic architecture.

It may not be out of place to mention, in connexion with the former and more general causes, a practice which is common in India, and which must, no doubt, exert a considerable degree of influence, in giving rise to a particular form of mania. We allude to the barbarous mode of treating parturient women. Col. Sykes, in his interesting tracts of the "Vital Statistics of India," published in 1846, states that with a view to shorten the stages of labour, the parturient woman is subjected to the coarsest and roughest usage. This violent treatment generally fails to produce the desired object, but I have no doubt that it must frequently give rise to cases of puerperal mania.

Dr. Wise draws a comparison between the number of cases occurring in India, and that which obtains in England, but we are inclined to believe that he has underrated the amount of insanity in Bengal. He observes :—

"Thus, let us compare the number of the insane with the census of the inhabitants ; and suppose that, in Ceylon, there are from 120 to 130 insane patients under treatment, and from 400 to 500 lunatics in the island. In the circle of Bengal districts, from which lunatics are sent to the Dacca asylum, 157·5, the average of four years, may be supposed to be under treatment, and probably from 2000 to 2500 is the actual number of lunatics. The following is an approximative comparison with the returns from England and Wales :—

| | Population. | Lunatics. | | Per Cent. |
|---|---|---|---|---|
| | | Under Treatment. | Probable Actual Number. | |
| Island of Ceylon . | 1,009,008 | 125 | 450 | ·00,446 |
| Dacca Circle . . . | 9,891,484 | 157·5 | 2000 | ·00,202 |
| England and Wales. | 17,905,831 | 13,400 | 13,400 | ·00,754 |

It would appear, however, from the returns of the various dispensaries established in India, through the benevolent exertions of Lord Auckland, that insanity occurs more frequently than the above tables would lead us to infer. The returns to which we allude are published in Col. Sykes' interesting tracts. We have made a selection, not only of maniacal, but of paralytic and other kindred affections, and it is extremely probable that amongst the very large number of cases of paralysis, many of the subjects were afflicted with some impairment or derangement of the mental faculties, which complication might have been overlooked in drawing up the returns, which are admitted by Col. Sykes to have been defective. Moreover, in a general inquiry of this sort, it is of the greatest importance to ascertain the existence and amount of every description of cerebral disease. With this view, we have made the following resumé of nervous affections, common to Bengal and the northern provinces of India:—

| Mania. | Epilepsy. | Paralysis. | Apoplexy. |
|---|---|---|---|
| 534 | 339 | 1482 | 130 |

Total number of nervous affections treated at *Dispensaries only* . . 2485

The returns were sent in half-yearly, and extended over a period of several years, but Colonel Sykes says, "unfortunately there were many omissions, and want of continuity;" hence, the aggregate we have given is *below* the mark, and it is probable that there exists a greater amount of insanity than Dr. Wise's statements would lead us to infer.

The following remarks, which Dr. Wise makes on the popular notions of insanity, and on the establishment of lunatic asylums in India, will be read with interest.

"The Hindus, like many other people, usually consider the insane and the deformed as divinely favoured and protected. 'The lamp,' of the madman is said 'to be out,' and the soul, at certain phases of the moon, is supposed to return to the great god *Mahadeva:* this is called 'the hour of folly.' Other rude nations suppose that certain forms of insanity are produced by devils, or evil-disposed spirits. The consequence of a belief in such fancies is, that while some have a strong prejudice against sending their relatives from home, to which they are supposed to bring prosperity, and particularly to an asylum where they are believed to be harshly treated; other families attach a degree of shame to insanity, especially should the patient be a female of good extraction, and there is, consequently, a desire to bury her existence in oblivion. It is therefore the wish of many to keep the insane members of a family in their own homes, unless when they become very troublesome. When the members of the family are poor, they usually neglect their insane relatives, who receive food from the charitable, and are generally allowed to wander about, until the disease is incurable, or until they have injured some one; when they are sent by the police to the district jail, where a ward was formerly appropriated for them

This plan has been changed, and capacious asylums for the insane have been formed in central positions in India, to which those of the neighbouring districts are sent. The Dacca asylum receives patients from ten districts, including Assam ; and they often arrive without the physician even knowing their names, or past habits ; or whether it is the first attack, of a few days' standing, or a confirmed and hopeless case of long duration. So that all statistical calculations must be considered as approximate rather than exact. Very few of their friends or relations will even take the trouble to furnish any particulars of the lunatics, so that little can in general be gleaned of their previous history, unless a few faint traces of the past may sometimes be obtained from the recollections of an individual, on his recovery."

The establishment of lunatic asylums is indeed a noble work of charity, and will confer greater honour on the names of our Indian rulers than the achievement of their proudest victories.

With respect to the influence of the seasons in producing insanity, Dr. Wise makes the following observations :—

"*Seasons.*—The greatest number of insane patients are brought to the asylum between the months of April and November, which embrace the hottest months of the year; the largest proportion of recoveries occurs during the cold months; and the most fatal months are from July to January, which embraces the most unhealthy season of the year. Hence we may conclude, that the great heat of the weather has an influence in producing insanity, so that when the hot weather occurs suddenly, the number of admissions is increased, and is diminished by a long course of cold weather, during which the system is invigorated."

We were rather startled to find our author expressing a superstitious belief in the influence of the moon ! He gravely states :—

"Careful observations have convinced me that, in the humid atmosphere of Bengal, the influence of the moon upon the paroxysms of insanity is considerable."

It seems the practice of *grinding* is not confined to the purlieus of our colleges. The following is a good example of the pernicious effects of *cramming* :—

"Mahammud Agem, a sickly youth, twenty years of age, became a disciple of a fanatic, and, under his guidance, was taught Persian and Arabic. The Koran and other works he learned by rote, so that his memory was fatigued without his understanding being enlightened: inheriting a weakness of the mind from the total want of intellectual culture of his parents, he became disturbed in his sleep—saw visions—and became alarmed by the appearance of devils, who threatened to punish him for not performing some fancied work. The studies being continued, he became insane, and was then sent to the asylum. He at first refused to eat, and milk was injected into his stomach. He soon improved in health, took much exercise,—and by the relaxation of the mind, change of residence, and the healthy atmosphere of the asylum, he soon got well."

Grief, as in this country, can number its victims. Many cases are instanced of derangement of mind from the bereavement of relatives, the failure of trade, and the loss of caste. Dr. Wise does not consider the abuse of spirituous liquors a very common cause of insanity, the priests being opposed to their excessive use, although the statements of Col. Sykes would lead us to an opposite conclusion. He, the Colonel, states that drunkenness is very rife in Patna, and that the town is surrounded with toddy trees, from which the natives are known to extract a highly intoxicating drink.

Our readers will, no doubt, feel interested in the following particulars relative to the use of those deleterious eastern luxuries—opium, gunjah, and churus.

"*Opium.*—Opium is very generally employed by the Mussulmans from its supposed property of lengthening life, and removing certain diseases, such as disorders connected with looseness, as diarrhœa, dysentery, cholera, and other discharges, as fluor albus, diabetes, watering of the eyes, coughs, etc. I have known an infant, a few months old, so habituated to the soothing influence of opium, that it required a supply every night to keep it quiet. It was given in this case to save trouble, and to strengthen, as it was supposed, the child, and prevent it suffering from the bad effects of cold, whereas it must have had quite an opposite effect. Such a habit in the adult produces great debility and emaciation, curtailing the enjoyments, and shortening the duration of the life of the individual. The effect of opium, when taken in large quantities, is succeeded by that painful longing, and most distressing irritability and weakness, which often destroy its votaries, by rendering them subject to other diseases, and sometimes unhinge the mind."

"*Gunjah.*—The use of the preparations of Indian hemp or gunjah (Cannabis Sativa), has a much more pernicious influence on the mental faculties than opium or spirits, which are more transitory in their effect. Gunjah was well known, and its effects understood for many ages, in the South of Africa, in America, and in the greater part of Asia. It appears to have been employed in the temples of the ancient Greeks for its intoxicating quality, and it is still employed for the same purpose by the Brahminical priests of India, and by the dissipated and depraved, more particularly of the lower class. With them it is supposed to be the ready agent to enable the person to bear hard and continuous labour without fatigue, to prevent the pain accompanying physical injury, to guard against insalubrious climates and unhealthy seasons. It likewise produces pleasing and cheerful intoxication, and has other qualities which lead to its deleterious use—as it kindles the imagination, inflames the sensual passions, and the appetite for food. Some who use it state that it renders them more fervid in their devotions, circulates the blood, and clears the voices of singers. But it is also well known that a constant, or large consumption of it, makes the person unfit for business, and, if continued, produces insanity.

"When gunjah is employed as a luxury, it is used in combination with prepared, or dry tobacco leaf; each pipe-full (chillim) being filled up with from two to eight annas' weight of the compound. Sometimes from twenty to thirty chillims are used daily. The cost is about one rupee and eight annas, the two pounds (Seer). Such is the quantity consumed, that three or four rupees a month are often spent by one individual on this deleterious drug; and such is the fascination, that to increase the gratification the smoke is often passed into the pharynx and nostrils, and after remaining some time it is discharged.

"It is customary for several of these miserable votaries to meet at one of their houses, and sit on the floor in a circle; each then takes a draught of the hookah, which has been prepared with gunjah and tobacco, and hands it to his neighbour. Intoxication soon occurs, as it is stated that four or five mouthfuls are sufficient to intoxicate persons, even accustomed to the use of the drug. In my inquiries, in the Dacca Insane Asylum, as to the cause of such persons' insanity, I found that of those who had formed the habit of using gunjah before admission into the asylum, and whose statement was confirmed in every case by relatives and friends, when that could be done, out of 286 that were in the asylum at the commencement, and were admitted during the year of report, seventy-seven, or nearly a third, had been rendered insane by the pernicious use of gunjah, to which the lower classes are so often habituated, from its agreeable intoxicating nature, and cheapness. The effects, however, are after a certain dose transient, and are soon followed by great debility.

"The remarkable effect of gunjah is, that it in an agreeable manner excites or modifies sensibility and combination of ideas, but it does not itself give origin to them. The enjoyment is entirely moral, and not like the gratification of the amatory passion. It, by use, weakens the animal passion, memory, the power of voluntary control of the thoughts, or fixing the attention. By a great effort the mind can, for a moment, be restored to its original powers. The gunjah creates an increase of appetite, a moderate exhilaration of spirits, sometimes an intense sensation of happiness. In other cases there is a weight of the head, and an uncomfortable sense of restlessness and palpitation of the heart. Occasionally the person exhibits a disposition to assume the recumbent position, and to bring the limbs and trunk together. In one case the patient took a poisonous dose given by an itinerant beggar, to ensure the good will of his neighbours. It produced intoxication and great heat of the body, which induced him to proceed to the river to bathe, when this disposition was so great that both his head and arms went under water, and he would have been drowned had he not been observed. In this case the single dose produced insanity, for which he was sent to the asylum; and it was three months before he was discharged as cured.

"These religious mendicants are a great curse to India. One day I asked one of them, a notorious gunjah-eater, what was his occupation. Placing his hand on his stomach, he said, 'Eating, and smoking gunjah.' The dreadful cannabis! 'But, what is your trade?' He added, 'To contemplate the Great God.' 'And where is your home?' He pointed downwards, and answered, 'In the earth.' The effects of gunjah are most pernicious. A young man, twenty-five years of age, was admitted on the 1st August, and discharged from the asylum on the 22nd, well. He was brought back in six days, much worse than he had been during his first attack. I found he had again indulged in gunjah, having taken five or six chillims daily. He recovered in three months; and requested to be allowed to remain in the asylum, as he could not resist the longing desire for gunjah, and dreaded the fearful consequences. This is the usual course of these unfortunate individuals. They get well, and return to the old habit, as soon as they leave the asylum, and either die, or are sent back. During the year of report, of the seventy-seven patients, twenty-six were cured, and five died from bowel complaints. The others remained in the asylum. Several had been more than once there. It must be allowed that there are other causes which, at the same time, aggravate the effects of the gunjah, such as exposure to the sun, bad food, unhealthy climate, and the irregular and bad habits of the individuals who are the chief sufferers."

"The difficulty of breaking the habit of using gunjah is always very great, and the recurrence of the insanity is generally the consequence even of a slight indulgence. Some years ago, I tried to prevent an unfortunate young man from obtaining his usual supply. He was reduced to a skeleton, took little or no food, and lived only to enjoy the fascinating drug. He was in a state of great nervous distress on the withdrawal of the gunjah, and complained bitterly of the privation: but no bad effects followed, and his health and strength improved while he remained in the hospital. In the Dacca jail it was found that the privation of the usual quantity of the drug and opium produced diarrhœa, etc., which was checked by allowing a small quantity daily."

"*Churus*, or the inspissated juice of the Indian hemp plant, is usually taken mixed with water. In half-an-hour it produces intoxication of a most cheerful kind, increases the appetite for food, and for sensual enjoyments. Should the Government restrict the sale of gunjah, many would be forced to give up the pernicious habit, as churus would be too expensive to enable them to purchase it."—page 14.

"The churus is prepared in Bengal by beating a quantity of the Indian hemp plant, exposing it to the influence of the night dew, and pressing the bruised plant with the naked hand, to which the churus adheres: it is then scraped off for use. It is of much higher price than the gunjah, and is more rarely used, particularly as it is far less stimulating, and produces a lighter degree of intoxication. So great is the difference, that ten pipes of churus will not cause the same effect that one of gunjah will produce. Still, churus is sometimes used by the rich, and by singers, etc."

We have again to regret the carelessness with which the author has drawn deductions from facts replete with physiological and pathological suggestions. It will be seen in the above extracts, that he flatly contradicts himself when speaking of the effects of the gunjah. In the first instance, he says, "it kindles the imagination, influences the sensual passions, and the appetite for food." He subsequently observes, "the enjoyment is entirely moral, and not like the gratification of the amatory passion." Dr. O'Shaughnessy and other writers on the Indian hemp, speak very decidedly as to its aphrodisiac properties. It would seem to act especially on the cerebro-spinal system, producing in small doses symptoms of inebriation of a cheerful character, followed by confusion of ideas, and lastly, sleep. In large doses it is a powerful narcotic. It is less certain in its operation than opium, but possesses advantages over the latter drug, inasmuch as it does not impair the appetite, but, on the contrary, increases the desire for food. Neither does it arrest the bronchial nor other secretions, as is the case with opium. We can speak favourably of the efficacy of the Indian hemp, in the treatment of some forms of insanity, accompanied with great debility, given with the view, not of producing a hypnotic or narcotic effect, which has hitherto been the practice, but as a *nervine stimulant.*

On the physical symptoms of insanity he offers nothing new, and his observations on the appearances found on dissection are comprised in the following very brief paragraph.

"On *dissection,* the skull of the insane is often very thick, and more serum than usual is found in the cavities of the brain, with marks of previous inflammation, such as a thickening and opacity of the arachnoid membrane, and often preternatural hardness of certain parts of the substance of the brain, with more or less alteration of the cortical substance."

The mortality amongst the patients at Dacca appears to have been very great, as will appear by the following statement :—

"*Mortality.*—The mortality among the patients in the Dacca asylum has always been very great; but subject to very considerable changes, from the peculiarities of the season, etc. During one year that an able predecessor of mine superintended the asylum, the deaths amounted, among the males, to 32 per cent.; and the cures, including cases relieved, and made over to their friends, to 51 per cent. of the admissions. Among the females, during the same period, the per centage was $45\frac{1}{2}$ cured, to $38\frac{1}{8}$ per cent. deaths."

Our author's remarks on the classification of the lunatics in the Dacca asylum, need not detain us, but the following affecting cases, one exhibiting an unusual degree of attachment between two lunatics, and the other relative to a melancholy instance of puerperal mania, will, no doubt, interest some of our readers :—

"*Kureem Khan*, æt. 39, lost some land, after much vexatious litigation, which, together with the free use of opium and probably gunjah, appeared to be the exciting cause of his insanity. He was admitted into the asylum in a sufficiently sane state to answer questions, and to agree to diminish the pernicious habit he had contracted of eating large quantities of opium. This was done by commencing with fifteen grains of solid opium, his usual daily quantity, and diminishing the dose by a grain daily, until none was left. He now appeared to feel no want of the drug. During the interval of the paroxysms, he was a strong intelligent man, and assiduously worked in the cook-room as an assistant, or in preparing screens, brooms, etc.

"He took charge of an insane orphan boy, who was brought to the asylum. This unfortunate child was found near the place where the Hindus burn their dead. He was very much emaciated from starvation, and was in a state of amentia, probably from ill treatment. What a story of cruelty might he have revealed, but this was denied him! His reason was quite gone—'his lamp was out.' But the broken reed was cherished, and supported by Kureem Khan; and it was an interesting sight to observe the care with which he attended to the helpless child, who crouched behind his protector, when any stranger approached; and then he would look up and smile upon his benefactor for placing him in security,—and it was such a smile of sweetness! His spare body supported one of the most beautiful heads I ever saw. Such a beauty of form!—such a brow!—such large black expressive eyes, sheltered behind such graceful eyelashes!—and such a beautiful chiselled mouth, as would have formed a study for Raphael! The foster father never left his child, and tried to instruct him. He carefully taught him to repeat scraps of Sadee; but such was the defect of the child's memory that he required continual prompting. His own name he did not recollect. Poor boy! gratitude appeared to be the only remnant of his ruined mind. His protector, when the paroxysm of insanity was on him, changed his whole manner; for some time he remained silent, and seemed to wander about to get rid of the inward distress that preyed upon him, and the insane child was the first to feel its effects; indeed, such was the acuteness of his instinct of danger, that, on the first indication of the coming paroxysm, he fled and hid himself from his violence. The madman's face assumed a most diabolical expression of rage; and he immediately went in pursuit of the boy, and his other enemies, who were now to be chastised. This he supposed he did by rolling up a portion of his clothes, and beating them with a stick, and heaping all kinds of abuse upon the bundle. In other cases he took a brick, or the trunk of a tree, and beat it; sometimes with a stick, with his hand, and with his elbow; and this was done with such violence as to bruise and injure his arm. On these occasions he changed his elbow for his feet, and struck at his clothes, or the brick he had procured, until quite exhausted. I once called him to me, when in this state. He told me a long list of grievances, of his riches, his villages, and his rank; which had all been taken from him by the base treachery of lawyers, and had reduced him to his present state. He then appeared quite satisfied, sought out the boy, and returned to his work. On one occasion this madman had a visit from his brother, and they remained for some time on most amicable terms, until something irritated him; when he suddenly became enraged, and beat his brother, so as to oblige him to fly for his life.

"To obtain ornaments for the insane boy was the great stimulus to exert himself, and he executed a good deal of work, to get money to purchase clothes and ornaments with which to decorate him. Again a paroxysm of insanity would occur, and the boy was obliged to hide himself; although a short time before he had decorated him with ornaments, promised to make him a landed proprietor, and called him his son and brother.

"These ornaments were generally stolen during the boy's sleep. At other times the rupees which the protector got for his work, were put into the hands of some one in the asylum, and so bad was his memory, and unjust his friends, that he was often cheated out of them. In other cases he seemed to accuse those arouud him falsely for having, he alleged, misappropriated his money.

"*Puerperal madness.*—The variety of mental derangement incident to women soon after parturition seems to be less common in Bengal than in Europe. A respectable Mohammedan requested me to see his daughter, fifteen years of age, who had become deranged after her first confinement. The visit was made in the evening, and the picturesque thatched house was surrounded by numerous majestic palms, plantains, bamboo clumps, and other beautiful tropical plants, which were partly illuminated by the setting sun. When we were seated, the afflicted female, dressed in a thin muslin dress, and guarded by several attendants, was brought and seated before us. During the interview she continued laughing, and chattering nonsense. Her parent, in his long Arab dress, and flowing beard, stood beside her, and related how she had been happily married, had lost her infant, and by the sudden stoppage of a dysenteric affection, had been reduced to her present state. 'She was the apple of my eye.' 'She could recite our prayers,' he said. 'None was equal to her in learning; but now she has forgotten all.' 'And I,' continued her father, 'was so fond and proud of her; and now she will not attend to me, or even eat of her favourite dishes. I prepared them for her yesterday, set them before her, and urged her to eat them for my sake; but she spurned them from her, abused me, and tore my face and clothes. Her loss to me is like the loss of repose: I shall never recover it.' The father stated these and other particulars with the minuteness of one intensely interested in the subject. His pale countenance and tearless eyes proved that his grief was passing show. He continued,—'She now knows her father no longer;' and, turning to me, he added,—'Now, I look to you as her father, and mother, and her only refuge. Your kindness in coming to visit my daughter shall receive my everlasting gratitude. She will not even eat any longer for me.'

"The treatment which was required in such cases was stated. Her fine hair was to be allowed to be cut off, with the exception of a central portion; but the husband was to be consulted, before the general plan of treatment was to be commenced. The father declared it would be a disgrace to the family—who lived in a hut, and found difficulty in procuring the means of subsistence—to remove the patient to the asylum, where she would receive the greatest attention and kindness, which would give her the best chance of recovery from her terrible malady. But such is the power of habit, and the weakness of human nature, among unenlightened races."

In the treatment of his patients, Dr. Wise appears to have exhibited judgment and humanity in carrying out the principles established by our most enlightened physicians. The utility of actively employing the insane, is well exemplified by the following facts, which serve to show that manual occupation is of advantage to *every* class of individual and not confined to those only who have been previously habituated to hard labour :—

"This humane system of treatment requires the constant vigilance of the superintendents. In India this cannot always be relied on; and we can never calculate that the patients are not neglected, or degraded by the attendants. There is a great difficulty in inducing patients, even though convalescent, to submit to any sort of labour. A rajah will pretend to be only able to act in ordering diplomatic matters, a landed proprietor in arranging accounts, a holy fukeer in contemplation, etc.; but we have seen that the rajah may become an excellent basketmaker, a landed proprietor may be employed with advantage in carrying water, and the fukeer in cleaning the wards in the morning. When the first sense of repugnance of the person, at such an employment, and their imaginary high rank is got over, the new occupation changes their morbid trains of thoughts, and thus acts very favourably on their diseased minds. A case occurs to me :—A middle-aged man was brought to the asylum in a state of amentia, and much weakened in bodily health. He declared he was the father of mankind; and, as the first man, he never wore clothes. At times he seemed silent, and would not work. After attention to his general health, he was taken to the workshop, and in a few days he began to work, of his own accord, and to wear clothes. He soon became quite altered, became very industrious, inoffensive, answered questions rationally, and improved in health. There was every prospect of his speedy recovery, when he was attacked with diarrhœa, and died. Thus, by perseverance, by example, and a little indulgence, especially in diet, and dress in females, their obstinacy will in general be removed; and an influence over the minds of such patients will be of great importance to their recovery."

In bringing our brief notice of this pamphlet to a conclusion, we must observe, that although our duty has constrained us to speak freely of the treatise under review; this has been done in no unfriendly spirit, but solely with the view of directing the writer's attention to the partiality of his views, as contrasting with the largeness and importance of the subject. We have shown our appreciation of his labours by the numerous extracts we have given from his work, and as the pioneer of future observers in this interesting field, we give him a brother's welcome.

---

### Art. IV.—RATIONALE OF INDUCTIVE EVIDENCE.*

